# Psychiatric morbidity among adult patients in a semi-urban primary care setting in Malaysia

**DOI:** 10.1186/1752-4458-3-13

**Published:** 2009-06-18

**Authors:** Ruzanna ZamZam, Maniam Thambu, Marhani Midin, Khairani Omar, Pervesh Kaur

**Affiliations:** 1 Department of Psychiatry, Faculty of Medicine, Universiti Kebangsaan Malaysia, Kuala Lumpur 56000, Malaysia; 2 Department of Family Medicine, Faculty of Medicine, Universiti Kebangsaan Malaysia, Kuala Lumpur 56000, Malaysia

## Abstract

**Background:**

Screening for psychiatric disorders in primary care can improve the detection rate and helps in preventing grave consequences of unrecognised and untreated psychiatric morbidity. This is relevant to the Malaysian setting where mental health care is now also being provided at primary care level. The aim of this paper is to report the prevalence of psychiatric illness in a semi-urban primary care setting in Malaysia using the screening tool Patient Health Questionnaire (PHQ).

**Methods:**

This is a cross-sectional study carried out in a semi-urban primary healthcare centre located south of Kuala Lumpur. Systematic random sampling was carried out and a total of 267 subjects completed the PHQ during the study period.

**Results:**

The proportion of respondents who had at least one PHQ positive diagnosis was 24.7% and some respondents had more than one diagnosis. Diagnoses included depressive illness (n = 38, 14.4%), somatoform disorder (n = 32, 12.2%), panic and anxiety disorders (n = 17, 6.5%), binge eating disorder (n = 9, 3.4%) and alcohol abuse (n = 6, 2.3%). Younger age (18 to 29 years) and having a history of stressors in the previous four weeks were found to be significantly associated (p = 0.036 and p = 0.044 respectively) with PHQ positive scores.

**Conclusion:**

These findings are broadly similar to the findings of studies done in other countries and are a useful guide to the probable prevalence of psychiatric morbidity in primary care in other similar settings in Malaysia.

## Introduction

Most of the psychiatric morbidity in the community is seen at the primary care level [1-5]. Studies using screening instruments have reported prevalence rates ranging from 16 to 43% of general practice attenders [1,6-8]. A study of psychiatric morbidity using the General Health Questionnaire (GHQ) reported rates up to 30% [1]. In a Malaysian primary health setting the prevalence of psychiatric morbidity using GHQ-30 item version was 26.7% [9]. In the general population, as measured by GHQ-12 in the Malaysian National Health and Morbidity Survey, the prevalence of psychiatric disorder was 11% [10].

It has been reported that primary care practitioners miss about one third of psychiatrically ill people [4,11,12]. A number of reasons have been adduced for this. Patients seeing their primary care doctors tend to somatize their emotional distress, presenting with physical symptoms rather than overt psychological symptoms [13]. Medical history is often taken in conditions of little privacy thereby discouraging patients from sharing sensitive aspects of their distress [6]. Primary care practitioners (PCP) may also not be confident in diagnosing and treating psychiatric disorders [14,15] Moreover PCP tend to have limited time in which to obtain a psychiatric history [15].

The consequences of psychiatric morbidity such as depression when it is not identified and treated can be severe. These include suicide, loss of jobs and relationships, and deterioration in physical health including higher risk of myocardial infarction [16-18]. Early detection is important because it would reduce not only the above mentioned consequences but also unnecessary suffering for the patients [19,20]. In view of the serious consequences of psychiatric morbidity it would be useful for us to explore the prevalence of psychiatric morbidity in our own local setting.

The use of self-report screening tools in primary care settings is potentially helpful in view of the impediments to recognition and treatment of mental disorder in primary care. A number of screening tools exist, including GHQ, Self-rating Questionnaire (SRQ) and the Patient Health Questionnaire (PHQ) [21-23]. The aim of this paper is to report the prevalence of psychiatric disorder in a semi-urban primary care setting using the PHQ.

## Methods

This is a cross-sectional study carried out in a semi-urban primary healthcare center located south of Kuala Lumpur. The center is a government run healthcare facility which is easily accessible by public transport. The center caters for the health needs of a population of 603,800 people of whom a majority are from the lower middle socioeconomic group. The center provides general medical outpatient care, emergency services and special clinics for antenatal, diabetes, hypertension, psychiatric illnesses and HIV-related diseases. The average attendance in a month is about 5,000 cases (100 to 150 patients per day), a majority of whom are antenatal cases seen by nurses and midwives. Other facilities include laboratory investigations, ECG, radiography and ultrasound. The clinics are run by a primary healthcare physician, one medical officer, eight nurses, two midwives, two health attendants, two assistant pharmacists and one laboratory technician.

Patients aged from 18–70 years were selected based on their registration number and subjected to a systematic random sampling. Exclusion criteria were patients who did not consent and those who were not literate.

### Instruments

The PHQ was translated into Bahasa Malaysia and back translated. It was pre-tested to ensure that the original meanings were retained. The questionnaire consists of items that enable identification of eight psychiatric diagnoses including somatoform disorder, major depressive disorder, panic disorder, binge eating disorder, bulimia nervosa, alcohol abuse, other anxiety disorders and other depressive disorders.

The PHQ was validated against the GHQ-30 which has previously been validated for a Malaysian population [24]. The results of the validation will be reported in a subsequent paper. The respondents were also requested to complete a data form consisting of sociodemographic data, psychiatric history and recent life events.

### Procedure

The patients completed the forms while waiting to see the doctor. Once they were in the consultation room, the doctor verified positive responses and applied the diagnostic algorithm. The time taken by respondents to complete the questionnaire was 5 to 15 minutes. The ethics approval for the study was obtained from the Research Committee of the Department of Family Medicine, National University of Malaysia.

## Results

The sociodemographic characteristics of the sample are shown in Table 1. A total of 267 subjects returned questionnaires during the study period. Four questionnaires were incomplete and were excluded from analysis. The sample consisted of 97 (36.9%) young adults aged 18 to 29 years, 91 (34.6%) aged 30 to 39 years, and 75 (28.5%) aged 40 to 70 years.

**Table 1:** Demographic data of respondents

Demographic Data		Respondents (n = 263)	
		
		No.	Percentage (%) against total
Age group	18 ~ 29 years	97	36.9
	30 ~ 39 years	91	34.6
	40 ~ 49 years	51	19.4
	50 ~ 59 years	15	5.7
	60 ~ 70 years	9	3.4

Sex	Female	188	71.5
	Male	75	28.5

Race/Ethnic	Malay	199	75.7
	Chinese	29	11.0
	Indian	31	11.8
	Others	4	1.5

Education Level	None/Primary	27	10.3
	Secondary	170	64.6
	College/Tertiary	66	25.1

Marital Status	Single	26	9.9
	Married	233	88.6
	Widow/Divorced	4	1.5

Occupation	Professional, tech. & rel. work	78	29.7
	Clerical & Sales	69	26.2
	Production workers & laborers	35	13.3
	Economically inactive	81	30.8

Income Group	≤ RM1, 000	39	14.8
	RM1, 001 ~ RM1, 999	121	46.0
	RM2, 000 ~ RM2, 999	57	21.7
	≥ RM3, 000	46	17.5

Clinical Data		Respondents (n = 263)	
		
		No.	Percentage (%) of total respondents

PHQ	Positive	65	24.7

GHQ	Positive	71	27.0

Previously known medical illness	Yes	52	19.8

Previous history of psychiatric illness	Yes	1	0.4

Family history of psychiatric illness	Yes	1	0.4

History of stresses in the last 4 weeks	Yes	18	6.8

History of menstrual problems	Yes	25	9.5

Of the 263 respondents who returned completed questionnaires, 233 (88.6%) were married. The majority of the respondents were female (n = 188, 71.7%). Malays formed the majority ethnic group (n = 199, 75.7%), followed by Indians (n = 32, 12%), Chinese (n = 29, 11%) and other races (n = 3, 2%).

Most of the respondents had at least secondary school education (64.6%) and 69% were employed. Almost half (46%) were from the lower income group.

About twenty percent had a previously known medical illness (including diabetes mellitus, hypertension, coronary heart disease or bronchial asthma). One respondent had previous psychiatric illness while another reported a family history of psychiatric illness (0.4%). Only 6.8% reported having a history of stress or losses in the previous four weeks.

It was found that 24.7% of respondents had at least one PHQ positive diagnosis. Some had more than one diagnosis. The diagnoses included depressive illness (n = 38,14.4%), somatoform disorder (n = 32, 12.2%), panic and anxiety disorders (n = 17, 6.5%), binge eating disorder (n = 9,3.4%), alcohol abuse (n = 6, 2.3%) (Table 2).

**Table 2:** Clinical data of respondents

PHQ Diagnosis		Respondents (n = 263)	
		
		No.	Percentage (%) of total respondents
Somatoform disorder	Positive	32	12.2

Major depression & other depressive Syndrome	Positive	38	14.4

Panic Syndrome & other anxiety syndrome	Positive	17	6.5

Binge eating disorder	Positive	9	3.4

Alcohol abuse	Positive	6	2.3

To analyze the association between PHQ positive status and sociodemographic and clinical characteristics, chi-square test was done with p < 0.05 taken as statistically significant.

Table 3 shows that age group was the only sociodemographic factor significantly associated with positive PHQ. It was significantly associated with young age group between 18 to 29 years. (p = 0.036)

**Table 3:** Association of demographic data with PHQ score

		Respondents (n = 263)		
		
Demographic Data		PHQ positive	PHQ negative	P
		(% of total respondents)	(% of total respondents)	
Age	18 ~ 29 years	25 (9.5)	72 (27.5)	
	30 ~ 39 years	17 (6.5)	74 (28.1)	**0.036**
	40 ~ 49 years	20 (7.6)	31 (11.8)	
	50 ~ 59 years	1 (0.4)	14 (5.3)	
	60 ~ 70 years	2 (0.8)	7 (2.7)	

Sex	Female	47 (17.9)	141 (53.6)	0.865
	Male	18 (6.8)	57 (21.7)	

Race/Ethnic	Malay	47 (17.9)	152 (53.6)	
	Chinese	5 (1.9)	24 (9.1)	0.230
	Indian	11 (4.2)	20 (7.6)	
	Others	2 (0.8)	2 (0.8)	

Education Level	None/Primary	8 (3.0)	19 (7.2)	0.822
	Secondary	41 (15.6)	129 (49.0)	
	College/Tertiary	16 (6.1)	50 (19.0)	

Marital Status	Single	7 (2.7)	18 (7.2)	0.472
	Married	56 (21.3)	188 (67.3)	
	Widow/Divorced	2 (0.8)	2 (0.8)	

Occupation	Professional, tech. & rel. work	18 (6.8)	60 (22.8)	
	Clerical & Sales	16 (6.1)	53 (20.2)	0.574
	Production workers & labourers	12 (4.6)	23 (8.7)	
	Economically inactive	19 (7.2)	62 (23.6)	

Income Group	≤ RM1, 000	8 (3.0)	31 (11.8)	
	RM1, 001 ~ RM1, 999	33 (12.5)	88 (33.5)	0.740
	RM2, 000 ~ RM2, 999	12 (4.6)	45 (17.1)	
	≥ RM3, 000	12 (4.6)	34 (12.9)	

Note: total % exceeds 24.7% because some patients have more than one diagnosis.

Having a history of stressors in the last four weeks was found to be significantly associated with PHQ positive (p = 0.044)

## Discussion

Screening for psychiatric disorders in primary care is an important step to improve services. It should prompt physicians to consider further full diagnostic interview and referral to specialized psychiatric services whenever necessary [5]

An earlier study [25] used the GHQ-30 in detecting psychiatric morbidity in an urban Malaysian primary care population. In this study the PHQ is used because it is designed to provide diagnoses of mental disorders in primary care [15,26]

In this study it was found that the prevalence of psychiatric disorder among adult patients attending a primary care center was 24.7%. This is similar to studies done in other countries which reported prevalence between 25% and 35% [2,27-29]. A study done in Taiwan showed a higher prevalence of 38.2% [30]

A previous Malaysian study of probable mental disorder in primary care, using GHQ, and carried out in an urban population attending private outpatient clinics in Kuala Lumpur, reported a prevalence of 29.9% [25].

The overall prevalence of psychiatric disorder (24.7%) and the prevalence of depressive illness (14.4%) are within the expected range in a primary care setting [2,27-29]. The prevalence of somatoform disorder (12.2%) and the lower than expected prevalence of anxiety disorders (6.5%) perhaps reflect the tendency of Asian patients to present their psychological distress in somatic symptoms [31-33]. An interesting finding from this study is a finding of the higher than expected prevalence of binge eating disorder (3.4%) which is, to the best of our knowledge, the first estimate of prevalence of this disorder in a primary care setting in Malaysia.

Other studies found that being female, unemployed, separated or divorced is associated with a higher probability of psychiatric disorder [1,2,4,10,29,34-36]. However in this study, the only two factors found to be associated with psychiatric morbidity were age between 18 to 29 and a history of recent stressors.

Inferences from this study should be drawn with caution because of several limitations. The questionnaire was validated against the GHQ and not against a structured clinical interview which would have given better data on psychometric properties of the PHQ. There was a preponderance of Malays and women in this sample rendering the findings not applicable to primary care settings with different ethnic composition. It is more representative of semi-urban primary care centers with a preponderance of Malay attenders from the lower and lower middle income groups.

Systematic random sampling was carried out thereby reducing sampling bias. The sample size was adequate.

## Conclusion

The healthcare centre in which the study was carried out shares common features with many other centres in rural Malaysia. Hence the study is potentially useful in estimating the prevalence of psychiatric disorder in similar settings.

## Competing interests

The authors declare that they have no competing interests.

## Authors' contributions

RZ, MM and MT were involved in writing of this article. KO and MT were also involved in planning and supervision of the study. PK was involved in data collection.
